# Phylogenetic Analysis of *Stenotrophomonas* spp. Isolates Contributes to the Identification of Nosocomial and Community-Acquired Infections

**DOI:** 10.1155/2014/151405

**Published:** 2014-04-10

**Authors:** Vinicius Godoy Cerezer, Silvia Yumi Bando, Jacyr Pasternak, Marcia Regina Franzolin, Carlos Alberto Moreira-Filho

**Affiliations:** ^1^Departamento de Pediatria, Faculdade de Medicina da Universidade de São Paulo, Avenida Dr. Enéas Carvalho Aguiar, 647-5° Andar, 05403-900 São Paulo, SP, Brazil; ^2^Hospital Israelita Albert Einstein, 05652-900 São Paulo, SP, Brazil; ^3^Laboratório de Bacteriologia, Instituto Butantan, 05503-900 São Paulo, SP, Brazil

## Abstract

*Stenotrophomonas* ssp. has a wide environmental distribution and is also found as an opportunistic pathogen, causing nosocomial or community-acquired infections. One species, *S. maltophilia*, presents multidrug resistance and has been associated with serious infections in pediatric and immunocompromised patients. Therefore, it is relevant to conduct resistance profile and phylogenetic studies in clinical isolates for identifying infection origins and isolates with augmented pathogenic potential. Here, multilocus sequence typing was performed for phylogenetic analysis of nosocomial isolates of *Stenotrophomonas* spp. and, environmental and clinical strains of *S. maltophilia*. Biochemical and multidrug resistance profiles of nosocomial and clinical strains were determined. The inferred phylogenetic profile showed high clonal variability, what correlates with the adaptability process of *Stenotrophomonas* to different habitats. Two clinical isolates subgroups of *S. maltophilia* sharing high phylogenetic homogeneity presented intergroup recombination, thus indicating the high permittivity to horizontal gene transfer, a mechanism involved in the acquisition of antibiotic resistance and expression of virulence factors. For most of the clinical strains, phylogenetic inference was made using only partial *pps*A gene sequence. Therefore, the sequencing of just one specific fragment of this gene would allow, in many cases, determining whether the infection with *S. maltophilia* was nosocomial or community-acquired.

## 1. Introduction

The genus* Stenotrophomonas* is genetically and phenotypically heterogeneous [1, 2], presently encompassing twelve recognized species validated in the List of Prokaryotic Names with Standing in Nomenclature (http://www.bacterio.cict.fr/s/stenotrophomonas.html).* S. maltophilia*, the genus type species, besides its biotechnological importance (nitrogen fixing, stimulation of plant growth), has gained clinical importance in the last two decades, being the only specie of the genus* Stenotrophomonas *found in the environment and also as an opportunistic human pathogen [1, 3, 4].* S. maltophilia* occurs in any aquatic/humid environment and is capable of colonizing and proliferating in abiotic surfaces, such as Teflon, glass, and plastics, and it has been isolated in niches as diverse as catheters, dialysis machine tubing, and drinking water reservoirs [4], causing nosocomial as well as community-acquired infections [4–7].

*S. maltophilia* can cause serious bloodstream and respiratory infections in immunocompromised patients, with reports on crude mortality rates between 18% and 69% (reviewed by Paez and Costa [8]).* S. maltophilia* infections have great relevance in pediatric hospitals, being associated to high morbimortality rates (crude mortality rates around 40%) in Intensive Care Unit (ICU): hospitalized and/or immunocompromised patients [9, 10]. Surveys from different geographic regions report an increasing rate of* S. maltophilia* infections in the last decade, probably associated with an escalation of invasive procedures, the spread of naturally carbapenen-resistant* S. maltophilia*,and the empirical use of broad spectrum antibiotics [3, 6, 10–12]. Furthermore,* S. maltophilia* is not exclusively a nosocomial pathogen, since it has been associated with community-acquired infections, mainly affecting patients with some kind of comorbidity [5, 7] and mostly related to water supply contamination [4]. In a recent survey of* S. maltophilia* bloodstream infections in Taiwan between 2008 and 2011 (153 cases), Chang et al. [7] found that 38.6% were community-onset (48.5% community-acquired and 52.8% healthcare-associated).

*S. maltophilia* has high-level intrinsic resistance to many unrelated antibiotics [4, 6]. Besides multidrug-efflux pumps and low outer membrane permeability, this bacterium can also acquire antibiotic resistance by horizontal transfer of resistance genes located on plasmids, transposons, and integrons [3, 4, 13–15]. The remarkable capacity of* S. maltophilia* for acquiring genetic factors of resistance to antibiotics and biocides shows the importance of conducting resistance profile and phylogenetic studies in clinical isolates, aiming to identify the origins of horizontal genetic transmission (environmental, nosocomial), and isolates with augmented pathogenic potential [4, 16].

Multilocus sequencing typing (MLST) has proven to be a reliable mean for inter- and intraspecies delineation of* Stenotrophomonas *spp. and a highly portable standard for strain characterization [2, 16]. Moreover, MLST provides an adequate window of discrimination for distinguishing among clusters of closely related isolates (clonal complexes) and, therefore, for unraveling horizontally transferred genetic information, because it modifies the composition inside clonal groups [16–19]. Here we used MLST and phylogenetic analysis, as well as biochemical and multidrug resistance profiles, for characterizing nosocomial isolates and clinical strains of* Stenotrophomonas* spp. and for identifying community- and hospital-acquired origin of nosocomial isolates.

## 2. Materials and Methods

### 2.1. *Stenotrophomonas *spp. Nosocomial Isolates and Strains

Twenty-nine nosocomial isolates obtained from Hospital Israelita Albert Einstein (HIAE), São Paulo, SP, Brazil; and thirteen clinical strains; five environmental strains; and five type strains (one clinical and four environmental) from different collections, in total 52 bacterial strains (Table 1), were selected for phylogenetic and phenotypic analyses for determining their phylogenetic profiles and association with biochemical and multidrug resistance characteristics. Fourteen of the HIAE nosocomial isolates were collected from patients hospitalized in the Intensive Care Unit (ICU). The clinical and environmental strains were obtained from the Belgian Co-ordinated Collections of Micro-organisms/Laboratorium voor Microbiologie of Universiteit Gent (BCCM/LMG, Gent, Belgium, http://bccm.belspo.be/index.php). The clinical strains were collected between 1976 and 1995 having different geographical origins. One of the five* Stenotrophomonas* type strains was obtained from the German Culture Deutsche Sammlung von und Mikroorganismen Zellkulturen GmbH (DSMZ Braunschweig, Germany) and the remaining four came from the BCCM/LMG culture collection.

Bacterial strains were preserved in Trypticase Soy Broth (TSB, Difco Laboratories, Detroit, Michigan) with glycerol (10% v/v) at −80°C. Nosocomial isolates were grown in TSB and, subsequently, these strains were cultivated on Trypticase Soy Agar (TSA, Difco Laboratories, Detroit, Michigan) to check for any eventual contamination. Belonging to the genus* Stenotrophomonas *was confirmed by sequencing of a 500 bp fragment of the 16S rRNA gene.

### 2.2. Biochemical and Drug Resistance Tests

Biochemical phenotyping of nosocomial isolates and bacterial strains was attained by using the API 20 NE kit (BioMérieux, Marcy-I'Etoile, France). Nitrogen-fixing capability—a common characteristic of environmental* Stenotrophomonas* strains—was determined for all the nosocomial and clinical strains here studied by means of the acetylene reduction assay, using the nitrogen fixing strain* Azospirillum brasilense* Sp7T as positive control [20].

The antibiotic resistance profiles of* Stenotrophomonas* nosocomial isolates and strains were determined by using susceptibility test discs [21, 22]. Bacteria were grown on Mueller-Hinton agar in the presence of the following antibiotic discs (Cefar Diagnóstica Ltda., São Paulo, Brazil): amoxicillin/clavulanic acid, 30 *μ*g; imipenem, 10 *μ*g; meropenem, 10 *μ*g; ceftazidime, 30 *μ*g; cefotaxime, 30 *μ*g; aztreonam, 30 *μ*g; ciprofloxacin, 5 *μ*g, levofloxacin, 5 *μ*g; chloramphenicol, 30 *μ*g, trimethoprim-sulfamethoxazole, 25 *μ*g; tetracycline, 30 *μ*g; and tobramycin, 10 *μ*g. The results were evaluated according to CLSI [21, 22].

### 2.3. Genomic DNA Extraction

Total genomic DNA of bacterial strains was extracted by using the Wizard Genomic DNA Purification Kit (cat. no. A1120, Promega Corporation, Madison, WI), according to the manufacturer's instructions. DNA quality and concentration were determined through 1.0% agarose gel electrophoresis using the Invitrogen Low Mass ladder (cat. no. 10068013, Invitrogen Corporation, Carlsbad, CA) and ethidium bromide staining and visualization under UV light.

### 2.4. Phylogenetic Analysis by Multilocus Sequence Typing (MLST)

Seven constitutive genes were chosen for phylogenetic analysis. Six of these genes—*atp*D,* gap*A,* gua*A,* pps*A,* nuo*D, and* rec*A—were firstly employed for inferring the population structure of the genus* Stenotrophomonas *by Kaiser et al. [16]. We also included the constitutive gene* rpo*A already used by our group [23].

Amplification conditions for* gap*A,* gua*A,* pps*A,* rec*A, and* rpo*A were 1X Taq DNA polymerase buffer, 1.5 mM MgCl_2_, 2 U of Taq DNA polymerase (cat. no. 10342020 Invitrogen Corporation, Carlsbad, CA), 25 mM of dNTPs, 10 mM of forward and reverse primers [16, 23], and water to adjust the final reaction volume to 50 *μ*L. The amount of DNA per reaction ranged from 20 to 40 ng depending on the size of the gene fragment to be amplified. The PCR reaction was performed for 40 cycles at the following temperatures: denaturation of DNA at 95°C/6 min, annealing at 62°C/15 sec, and extension at 72°C/1 min and 15 sec. A final extension was performed at 72°C/7 min. In order to amplify the sequences of* atp*D and* nuo*D [16], the annealing temperatures were decreased to 60°C and 48°C, respectively. The amplified products were purified with GFX PCR DNA and Gel Band Purification Kit (cat. no. 27-9602-01, GE Healthcare, Buckinghamshire, UK), following the manufacturer's instructions.

The sequencing reactions were performed using the Big Dye Terminator v3.1 Cycle Sequencing Kit (catalog no. 4337455, Applied Biosystems, Austin, Texas) and precipitated with the Big Dye XTerminator Purification Kit (catalog no. 4376486, Applied Biosystems, Austin, TX) following the manufacturer's instructions. The cycling temperatures were 95°C/20 sec for denaturing, 50–55°C/15 sec for annealing, and 60°C/1 min for elongation. This cycle was repeated 30 times. The sequences were read in the ABI 3500 Genetic Analyzer (Applied Biosystems, Forest City, California).

Sequence quality was analyzed and consensus sequences were identified by using the software Chromas Pro version 1.5 (Technelysium Pty Ltd, http://www.technelysium.com.au/chromas.html). After obtaining the consensus sequences for each bacterial strain, these sequences were exported in FASTA format for phylogenetic inference using the software MEGA 5 [24]. The phylogenetic trees were constructed by the neighbor-joining (NJ) method [25, 26], based on the p-distance [27, 28].

The GenBank (http://www.ncbi.nlm.nih.gov/genbank/) accession numbers for gene nucleotide sequences with 200 bp or more are* atp*D, KC209168-KC209214;* rec*A, KC209215-KC209254;* pps*A, KC209255-KC209300;* gap*A, KC209301-KC209345;* nuo*D, KC209346-KC209384; and* gua*A, KC209385-KC209424. These data were publicly released by GenBank on February 17, 2013.

Analysis of nucleotide sequence diversity was performed for the nosocomial isolates, clinical strains, and type strains using the software DNAsp [29] (DNA Sequence Polymorphism version 4.10) for* atp*D,* gap*A,* gua*A,* nuo*D,* pps*A,* rec*A, and* rpo*A genes. The parameter Pi (nucleotide diversity) corresponds to the average number of nucleotide differences per site between two sequences [30, 31] and its sampling variance [30].

## 3. Results

### 3.1. Biochemical and Drug Resistance Tests

All bacterial strains, with the exception of the four environmental type strains, five well-characterized environmental strains from LMG collection (see Section 2), three nosocomial isolates, and eight clinical strains were tested biochemically and identified as belonging to the genus* Stenotrophomonas*, what was confirmed for all clinical and nosocomial isolates by 16S rRNA gene sequencing. Theresults showed extensive biochemical similarity between these isolates (see Table 2). Moreover, all nosocomial isolates and clinical strains were found to be unable to reduce acetylene, revealing their incapacity for fixing nitrogen.

The 42 bacterial strains tested for drug resistance (Table 3) showed sensitivity to levofloxacin. The sensitivity to other antimicrobials was slightly or considerably lower: chloramphenicol (97.6%), trimethoprim-sulfamethoxazole (90.5%), ciprofloxacin (88.1%), ceftazidime (76.2%), tetracycline (71.4%), and tobramycin (71.4%). The isolates were resistant to imipenem (97.6%), cefotaxime (95.2%), aztreonam (85.7%), amoxicillin/clavulanic acid (85.7% each), and meropenem (81%). Interestingly, the majority of clinical isolates studied here show resistance to various beta-lactams (AMC, IPM, MER, CAZ, CTX, and ATM), a characteristic of clinical isolates of* S. maltophilia* [32, 33].

### 3.2. MLST Phylogenetic Analysis

In order to conduct phylogenetic studies using MLST data, fragments of seven constitutive genes were amplified and sequenced for 45 strains and for 7 strains were obtained from GenBank [2, 16] as indicated in Table 1. After sequence alignment, fragments sized 136 to 401 nucleotides for the* atp*D,* gap*A,* gua*A,* nuo*D,* pps*A,* rec*A and* rpo*A genes were used for nucleotide diversity and phylogenetic analyses. The gene with the highest number of polymorphic sites (87 sites) was* gua*A, followed by* rec*A (66 sites), and the genes* rpo*A,* pps*A and* gap*A had the higher rates of nucleotide diversity (Table 4).

**Figure 1:**
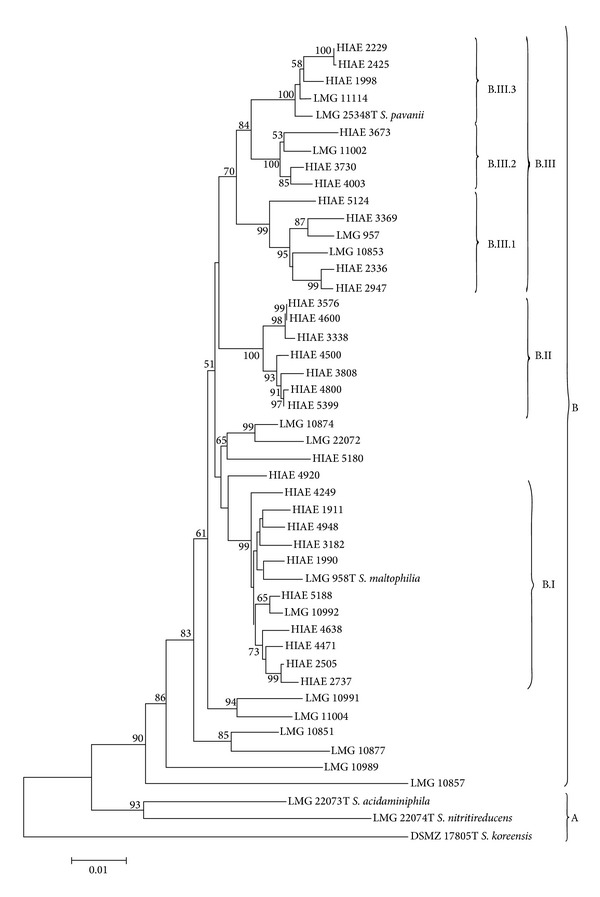
Neighbor-joining phylogram of 47* Stenotrophomonas *spp. clinical strains based on 7 concatenated genes. Neighbor-joining phylogram (1000 bootstrap replications, p-distance model) derived from concatenation of* atp*D,* gua*A,* gap*A,* nuo*D,* pps*A,* rec*A, and* rpo*A gene sequences. Phylogenetic groups and subgroups are indicated by A, B, B.I, B.II, B.III, B.III.1, B.III.2, and B.III.3. The numbers at branch nodes represent bootstrap values ≥50.

**Figure 2:**
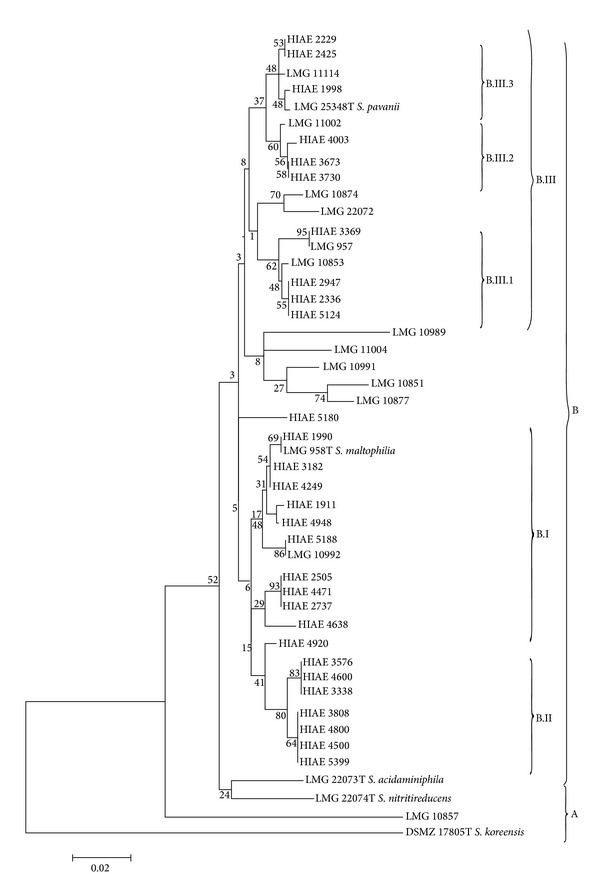
Neighbor-joining phylogram of 47* Stenotrophomonas *spp. clinical strains based on* pps*A gene fragment. Neighbor-joining phylogram (1000 bootstrap replications, p-distance model) for the gene* pps*A. Phylogenetic groups and subgroups are indicated by A, B, B.I, B.II, B.III, B.III.1, B.III.2, and B.III.3 similarly to the concatenated tree depicted in Figure 1. The numbers at branch nodes represent the bootstrap values.

**Figure 3:**
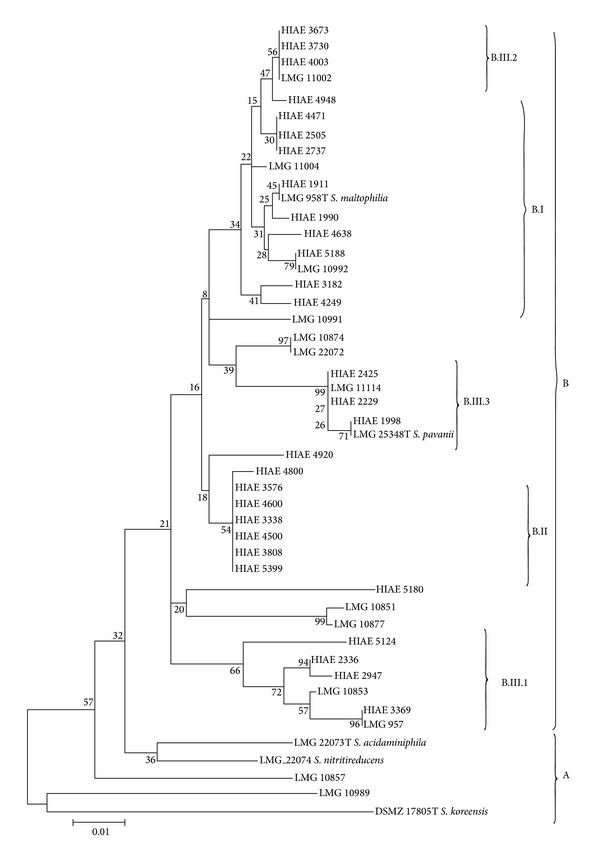
Neighbor-joining phylogram of 47* Stenotrophomonas *spp. clinical strains based on* rec*A gene fragment. Neighbor-joining phylogram (1000 bootstrap replications, p-distance model) for the gene* rec*A. The phylogenetic groups and subgroups are indicated by A, B, B.I, B.II, B.III, B.III.1, B.III.2, and B.III.3 similarly to the concatenated tree depicted in Figure 1. Note the shuffling between B.III clusters. The numbers at branch nodes represent bootstrap values.

Concatenated sequences were firstly obtained using fragment sequences of* atp*D,* gap*A,* gua*A,* pps*A,* nuo*D,* rec*A, and* rpo*A from 47 bacterial samples. These concatenated sequences were utilized to construct a neighbor-joining tree (based on p-distance). The phylogram (Figure 1) shows the division of these 47 strains in two groups: group A contains three type strains of environmental species of the genus* Stenotrophomonas* and group B includes all nosocomial strains and type strains of* S. maltophilia* and* S. pavanii*. Interestingly, group B is divided into three major subgroups (bootstrap > 70): B.I and B.II sharing high phylogenetic homogeneity and subgroup B.III, more heterogeneous, encompassing three clusters of strains (bootstrap > 99). Phylograms were also constructed using one-gene fragments (*pps*A or* rec*A genes). The* pps*A phylogram (Figure 2) shows a tree topology very similar to phylogram based on seven genes concatenated as depicted in Figure 1. On the other hand, the* rec*A phylogram (Figure 3) shows a shuffling between the B.III clusters and B.I subgroup, thus favoring the hypothesis of intersubgroup recombination. It is worth to note that groups B.I and B.III comprise isolates and strains with higher resistance to antibiotics.

### 3.3. Phylogenetic Analysis Including Environmental Strains of* S. Maltophilia*

**Figure 4:**
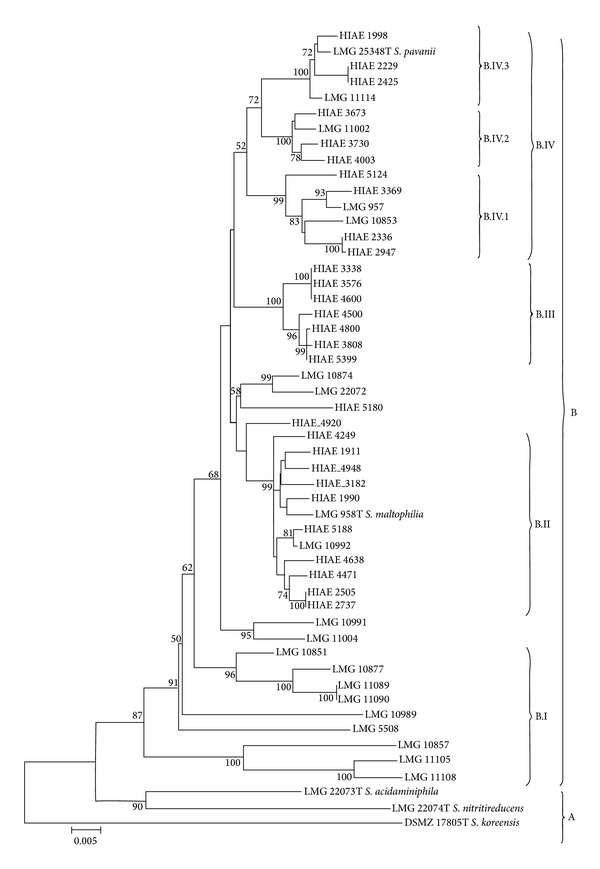
Neighbor-joining phylogram of 52* Stenotrophomonas *spp. clinical and environmental strains based on 6 concatenated genes. Neighbor-joining phylogram (1000 bootstrap replications, p-distance model) derived from concatenation of* atp*D,* gua*A,* gap*A, nuoD,* pps*A, and* rec*A gene sequences. Phylogenetic groups and subgroups are indicated by A, B, B.I, B.II, B.III, B.IV, B.IV.1, B.IV.2, and B.IV.3. The numbers at branch nodes represent bootstrap values ≥50.

A phylogram for analyzing the genetic similarity between clinical and environmental isolates of the genus* Stenotrophomonas* (all strains of Table 1) was built using the concatenated fragment sequences of the genes* atp*D,* gap*A,* gua*A,* pps*A,* nuo*D, and* rec*A (Figure 4). In this phylogram, it is possible to observe the formation of two groups of strains with higher phylogenetic proximity: group A contains only three environmental type strains of* Stenotrophomonas* and Group B contains all the other clinical and environmental strains plus the type strains of* S. maltophilia* and* S. pavanii*. Considering the phylogenetic proximities, group B can be divided in four subgroups (B.I to B.IV). B.I includes the five environmental strains of* S. maltophilia* and four nosocomial strains. B.II contains the type-strain of* S. maltophilia* and 12 nosocomial strains. B.III encompasses seven nosocomial isolates. B.IV contains the type-strain of* S. pavanii* and 14 nosocomial strains. The subgroup B.IV is subdivided into three small clusters: B.IV.1 and B.IV.2 encompass only nosocomial strains and B.IV.3 encompasses nosocomial strains and the type-strain of* S. pavanii*.

It is important to note that the subgroups and clusters B.II, B.III, B.IV.1, B.IV.2, and B.IV.3 have high phylogenetic similarity (bootstrap values between 99 and 100). Moreover, most of the strains in the groups B.II, B.III, B.IV.1, and B.IV.2 exhibit some intragroup metabolic differences, whereas the samples grouped into B.IV.1 show the same biochemical profile (Table 2). This phenotypic divergence between strains and isolates belonging to the same phylogenetic group indicates multiplicity of origin, as will be discussed later. Finally, the phylogram depicted in Figure 4 also shows that the majority of nosocomial isolates and clinical strains are phylogenetically distinct from environmental strains of* S. maltophilia*.

## 4. Discussion

This study adopted a phylogenetic approach—based on analysis of nucleotide sequences of fragments of specific genes by constitutive multilocus sequencing typing (MLST)—to investigate the clonal variability of nosocomial isolates and clinical strains of the genus* Stenotrophomonas*, which were also characterized biochemically and for their antibiotic resistance profile. This study allowed us to correlate the phylogenetic and phenotypic profiles with a multiclonal origin, reflecting the process of adaptability of bacteria of the genus* Stenotrophomonas* to different habitats.

A common phenotypic trait of all clinical strains and nosocomial isolates of* Stenotrophomonas* spp. studied in this work is their incapacity for nitrogen fixation. Biological nitrogen fixation is typical of environmental* S. maltophilia* (as well as of other* Stenotrophomonas* species) and depends on the nitrogenase structural gene* nifH* [21, 34, 35]. The expression of* nif* genes is controlled by nitrogen availability or the energetic status of the bacterial cell [36]. Therefore, one is tempted to hypothesize that the loss of the* nifH* gene cluster [37] could be an energy-saving adaptive event favoring the transition from free-living to opportunistic pathogen phenotype.

Phylogenetic analysis by MLST clearly showed that the nosocomial isolates and clinical strains of* Stenotrophomonas *spp. here studied have multiclonal origin and that the nosocomial isolates are grouped separately from environmental strains of the genus* Stenotrophomonas* (excepting* S. pavanii*). The pattern here observed is that of clonal complexes: groups are closely related, but not identical, to probable origin in a relatively recent common ancestor [16–18].

The phylogenetic groups and subgroups (see Figures 1 and 4) show high values (higher than 80) of bootstrap, indicating that amount of genetic variability here analyzed was adequate for defining five clonal groups (in Figure 1: subgroups B.I; B.II; B.III.1, 2, and 3 or in Figure 4: subgroups B.II and B.IV. 1, 2, and 3) among the clinical samples included in this study. Importantly, the 29 clinical samples isolated from HIAE patients of which 14 were collected from ICU patients (see Table 1) between August 2005 and July 2006 and 13 strains of clinical LMG, isolated at different times (between 1976 and 1995) and with distinct geographical origins, are positioned inside the subgroups B.I and B.III or B.II and B.IV identified in the phylograms shown in Figure 1 or Figure 4, respectively. Altogether, these data are consistent with a scenario of community-origin infections.

The multiclonal origin of clinical strains and nosocomial clinical isolates studied here is consistent with the characteristics of emerging and opportunistic pathogen described for* S. maltophilia* [4]. Moreover, this characterization is supported by detection of multidrug resistance in all these isolates and strains, what is a distinctive property of opportunistic pathogens of environmental origin [1, 4]. In the case of this study, it is worth to note that the majority of clinical isolates showed resistance to beta-lactams, which is typical of clinical isolates of* S. maltophilia* [11, 32, 33].

It is also interesting to note that some strains and clinical isolates with large phylogenetic proximity, with bootstrap values between 99 and 100 (see Figure 4 and Table 2), which could be considered as belonging to the same clonal group, do not share full identity in the metabolic profile, which indicates the community origin of these isolates and adaptive plasticity of its genome.

Comparative analysis of the phylograms based on* pps*A (Figure 2) and* rec*A (Figure 3) genes indicates the occurrence of intergroup genetic recombination involving subgroup B.III: there is clear separation between subgroups B.I and B.III in the phylogram generated from* pps*A, but there is an overlapping of B.I subgroup and part of samples of B.III.2 and B.III.3 subgroups in the phylogram generated from* rec*A. This result suggests the occurrence of a mechanism of horizontal transfer of genetic material that may occur by the insertion of phage, plasmids, pathogenic islands, or action of transposons [18, 38]. Moreover, it is well established that* S. maltophilia* can acquire genes involved in the resistance to antimicrobial agents and antibiotics from other environmental bacteria through horizontal gene transfer [4, 14].

Horizontal gene transfer events can modify the composition of clonal groups as evidenced by studies of MLST [16–19]. This is precisely the case of the isolates that integrate the B.I and B.III subgroups. This finding by MLST analysis indicates that the genome of subgroup B.III would be permissive to gain new factors of virulence and of resistance to antimicrobial agents. In fact, strains of the subgroups B.I and B.III are among the most multiresistant to antibiotics in this study (see Table 3).

The constitutive genes* gua*A,* gap*A,* pps*A, and* rpo*A showed the highest nucleotide variability among the seven genes selected for phylogenetic analysis by MLST (Table 4). Using data from sequencing of specific fragments of these genes, four phylograms were generated and the phylogram generated from* pps*A gene fragment sequence showed the discrimination of clonal groups closest to the concatenated phylogram shown in Figure 1. This result has practical interest since it indicates that the sequencing of a fragment of 257 bp of the* pps*A gene can serve to discriminate between clonal groups of isolates of the genus* Stenotrophomonas*. This simplified scheme can be employed in a clinical laboratory to check whether the infection with* S. maltophilia* was nosocomial or community-acquired.

## 5. Conclusions

The results of this work show that phylogenetic analysis by MLST is an important tool for the investigation of the genus* Stenotrophomonas* as an emerging pathogen. Such analysis is appropriate to identify the nosocomial or community origin of infections, serving to detect outbreaks and allowing a study of the population structure of the pathogen that is important for molecular epidemiology [16–18]. Moreover, studies using MLST are useful to investigate the origin of community infections by* S. maltophilia* and identify intermediate hosts, an area that remains unclear [4]. As emphasized by Maiden [17], MLST provides universal and rapid means for the identification of clonal complexes corresponding to hyperinvasive strains while they are spreading globally.

## Figures and Tables

**Table 1:** Nosocomial isolates, clinical and environmental strains, and type strains of *Stenotrophomonas* spp.

Strains	Source
Nosocomial strains	
HIAE 1911, HIAE 1990, HIAE 1998, HIAE 2336, HIAE 2425, HIAE 2737, HIAE 2947, HIAE 3182, HIAE 3338, HIAE 3576, HIAE 3730, HIAE 3808, HIAE 4003, HIAE 4249, HIAE 4471, HIAE 4500, HIAE 4600, HIAE 4638, HIAE 4800, HIAE 4920, HIAE 4948, HIAE 5124, HIAE 5399^U^	Tracheal secretion
HIAE 2229, HIAE 2505	Drain secretion
HIAE 5180	Leg ulcer
HIAE 3369	Human blood culture
HIAE 5188	Eye secretion
HIAE 3673	Brochoalveolar lavage
Clinical strains	
LMG 957, LMG 10851, LMG 10857*, LMG 10874*, LMG 11114	Human blood culture
LMG 10853*	Tracheal secretion
LMG 10877*	Patient suffering from sinusitis, pus
LMG 10989	Bile
LMG 10991*	Leg, pus
LMG 10992	Expectoration
LMG 11002	Mamilla
LMG 11004	Urine
LMG 22072	Cerebrospinal fluid
Environmental strains	
LMG 11089, LMG 11090	*Cichorium intybus* rhizosphere
LMG 11105, LMG 11108	Triticum roots
LMG 6608	rhizosphere
Type strains	
LMG 958 *S. maltophilia *	Cortical necrosis
LMG 22073 *S. acidaminiphila *	Mud
LMG 22074 S. nitritireducens*	Laboratory scale filter
LMG 25348 *S. pavanii *	Sugar cane
DSMZ 17805 S. koreensis*	Compost (straw + manure)

*Genomic sequences deposited in the GenBank were utilized in addition to sequences obtained in our laboratory for the genomic analysis by MLST. ^U^Strains collected in the ICU of Hospital Israelita Albert Einstein (HIAE).

**Table 2:** API 20 NE biochemical profile of *Stenotrophomonas* spp. phylogenetically close subgroups shown in Figure 1 or Figure 4.

Phylogenetic groups		Strains	Biochemical profile			
Figure 1	Figure 4		NO_3_	ADH	URE	PNPG
B.III.3	B.IV.3	HIAE 1998	−	−	−	+
		HIAE 2229	−	−	−	+
		HIAE 2425	−	−	−	+
		LMG 11114	−	−	−	+

B.III.2	B.IV.2	HIAE 3673	+	+	−	+
		LMG 11002	−	−	+	−
		HIAE 3730	−	−	−	−
		HIAE 4003	+	−	−	+

B.III.1	B.IV.1	HIAE 5124	−	+	−	+
		HIAE 3369	+	+	−	+
		LMG 957	+	−	−	+
		LMG 10853	−	−	+	+
		HIAE 2336	−	−	−	+

B.II	B.III	HIAE 3338	−	−	−	+
		HIAE 3576	−	−	−	−
		HIAE 4600	+	−	−	+
		HIAE 4500	−	−	−	+
		HIAE 4800	−	−	−	+
		HIAE 3808	−	−	−	+
		HIAE 5399	−	−	−	+

B.I	B.II	HIAE 4920	−	−	−	−
		HIAE 4249	−	−	−	−
		HIAE 1911	−	−	−	+
		HIAE 4948	−	−	−	+
		HIAE 3182	+	−	−	+
		HIAE 1990	−	−	−	−
		LMG 958	+	−	−	+
		HIAE 5188	+	−	−	+
		LMG 10992	+	−	+	−
		HIAE 4638	+	−	−	+
		HIAE 2505	−	−	−	−
		HAIE 2737	−	−	−	−

NO_3_: Potassium nitrate, ADH: L-arginine, URE: urea, and PNPG: 4-nitrophenyl *β*-D-galactopyranoside.

All strains were negative for the following: TRP: L-tryptophane, GLU: D-glucose (fermentation), ARA: L-arabinose, MAN: D-mannitol, GNT: potassium gluconate, CAP: capric acid, ADI: adipic acid, PAC: phenylacetic acid, and OX: oxidase and positive for the following: ESC: esculin, GEL: gelatin, GLU: D-glucose (assimilation), MNE: D-mannose, NAG: N-acetyl-glucosamine, MAL: D-maltose, MLT: malic acid, and CIT: trisodium citrate.

Strains LMG 25348, HIAE 2947, and HIAE 4471 were not tested.

**Table 3:** Antibiotic resistance profile of *Stenotrophomonas* spp. strains. Phylogenetic subgroups are indicated (Figure 1 or Figure 4).

Phylogenetic groups		Strains	Drugs tested: inhibition halo diameter in mm/resistance level												MR
Figure 1	Figure 4		AMC	IPM	MER	CAZ	CTX	ATM	CIP	LVX	CHL	SXT	TET	TOB	
B.III.3	B.IV.3	HIAE 1998	**0/R**	**0/R**	**0/R**	*17/I *	**0/R**	**0/R**	*17/I *	20/S	22/S	23/S	19/S	**0/R**	+
		HIAE 2229	**0/R**	**0/R**	**0/R**	22/S	**11/R**	**0/R**	22/S	30/S	26/S	29/S	22/S	15/S	+
		HIAE 2425	**0/R**	**0/R**	**0/R**	25/S	**12/R**	**0/R**	29/S	33/S	29/S	25/S	*18/I *	15/S	+
		LMG 11114	**0/R**	**0/R**	**0/R**	25/S	15/I	**0/R**	28/S	33/S	29/S	21/S	21/S	**0/R**	+

B.III.2	B.IV.2	HIAE 3673	**0/R**	**0/R**	**0/R**	22/S	**9/R**	**0/R**	23/S	30/S	20/S	26/S	20/S	23/S	+
		LMG 11002	30/S	**0/R**	28/S	36/S	18/I	**0/R**	32/S	28/S	34/S	34/S	20/S	28/S	+
		HIAE 3730	**0/R**	**0/R**	**0/R**	*16/I *	**0/R**	**0/R**	27/S	33/S	30/S	27/S	*17/I *	15/S	+
		HIAE 4003	**0/R**	**0/R**	**0/R**	27/S	*15/I *	**0/R**	29/S	33/S	28/S	31/S	25/S	25/S	+

B.III.1	B.IV.1	HIAE 5124	**10/R**	**8/R**	**13/R**	28/S	*16/I *	**0/R**	36/S	36/S	26/S	30/S	24/S	26/S	+
		HIAE 3369	**12/R**	**0/R**	**13/R**	30/S	*19/I *	**0/R**	31/S	35/S	28/S	24/S	*16/I *	23/S	+
		LMG 957	**11/R**	**0/R**	**10/R**	32/S	20/S	**0/R**	31/S	28/S	21/S	34/S	*17/I *	21/S	+
		LMG 10853	18/S	**0/R**	20/S	40/S	*21/I *	**0/R**	30/S	33/S	32/S	27/S	26/S	22/S	+
		HIAE 2336	**10/R**	**0/R**	**0/R**	*16/I *	**0/R**	**0/R**	**15/R**	21/S	22/S	20/S	**0/R**	25/S	+

B.II	B.III	HIAE 3338	**11/R**	**0/R**	**12/R**	28/S	**12/R**	22/S	27/S	32/S	29/S	28/S	20/S	24/S	+
		HIAE 3576	**10/R**	**0/R**	22/S	25/S	*17/I *	*16/I *	28/S	29/S	26/S	17/S	*17/I *	23/S	+
		HIAE 4600	**12/R**	**0/R**	**11/R**	26/S	**0/R**	23/S	30/S	33/S	32/S	24/S	21/S	22/S	+
		HIAE 4500	**0/R**	**0/R**	**0/R**	*17/I *	**0/R**	**0/R**	27/S	33/S	27/S	29/S	25/S	20/S	+
		HIAE 4800	**0/R**	**0/R**	**0/R**	31/S	**0/R**	**0/R**	28/S	31/S	21/S	27/S	28/S	20/S	+
		HIAE 3808	**9/R**	**0/R**	**0/R**	20/S	**0/R**	**0/R**	28/S	29/S	28/S	24/S	22/S	18/S	+
		HIAE 5399	**0/R**	**0/R**	**0/R**	*17/I *	**0/R**	**0/R**	29/S	30/S	36/S	25/S	23/S	22/S	+

B.I	B.II	LMG 22072	**0/R**	**0/R**	**0/R**	**0/R**	**10/R**	**0/R**	23/S	26/S	23/S	25/S	19/S	16/S	+
		HIAE 5180	**0/R**	**0/R**	**0/R**	31/S	**12/R**	**0/R**	23/S	33/S	34/S	31/S	33/S	**12/R**	+
		HIAE 4920	**11/R**	**0/R**	**0/R**	**0/R**	**0/R**	**0/R**	**0/R**	31/S	31/S	21/S	19/S	25/S	+
		HIAE 4249	**10/R**	**0/R**	**0/R**	21/S	**0/R**	**0/R**	**9/R**	17/S	29/S	**0/R**	**13/R**	**0/R**	+
		HIAE 1911	**8/R**	**0/R**	**0/R**	20/S	**0/R**	**0/R**	22/S	26/S	30/S	17/S	**10/R**	**0/R**	+
		HIAE 4948	**0/R**	**0/R**	**0/R**	31/S	**0/R**	**0/R**	28/S	31/S	21/S	27/S	22/S	**0/R**	+
		HIAE 3182	**0/R**	**0/R**	**0/R**	18/S	**10/R**	**0/R**	*20/I *	17/S	23/S	24/S	19/S	**0/R**	+
		HIAE 1990	**0/R**	**0/R**	**0/R**	**10/R**	**0/R**	**0/R**	26/S	25/S	27/S	**0/R**	**13/R**	**12/R**	+
		LMG 958 *S. maltophilia *	24/S	**0/R**	23/S	29/S	*15/I *	23/S	36/S	36/S	34/S	26/S	27/S	18/S	−*
		HIAE 5188	18/S	**0/R**	**0/R**	28/S	**10/R**	**0/R**	38/S	40/S	30/S	38/S	26/S	18/S	+
		LMG 10992	**0/R**	**0/R**	**0/R**	22/S	**13/R**	**0/R**	27/S	31/S	31/S	26/S	23/S	15/S	+
		HIAE 4638	**0/R**	**0/R**	**0/R**	21/S	**0/R**	**0/R**	23/S	27/S	23/S	21/S	21/S	23/S	+
		HIAE 2505	**0/R**	**0/R**	**0/R**	**10/R**	*15/I *	**0/R**	36/S	36/S	25/S	26/S	24/S	**0/R**	+
		HIAE 2737	**12/R**	**0/R**	**0/R**	*16/I *	**0/R**	**0/R**	29/S	32/S	34/S	27/S	*18/I *	**0/R**	+

B	B.I	LMG 11004	**0/R**	**0/R**	**0/R**	24/S	**10/R**	**0/R**	30/S	34/S	**0/R**	**0/R**	22/S	25/S	+
		LMG 10991	**10/R**	**0/R**	32/S	28/S	**10/R**	**32/S**	38/S	32/S	30/S	38/S	24/S	34/S	+
		LMG 10851	**0/R**	**0/R**	**0/R**	26/S	**13/R**	**0/R**	34/S	40/S	36/S	28/S	*16/I *	38/S	+
		LMG 10877	**0/R**	**0/R**	**0/R**	20/S	**0/R**	**0/R**	26/S	29/S	28/S	27/S	22/S	25/S	+
		LMG 10989	*16/I *	**8/R**	24/S	26/S	**12/R**	24/S	32/S	40/S	30/S	28/S	30/S	**8/R**	+
		LMG 10857	**0/R**	**0/R**	**0/R**	29/S	**0/R**	**0/R**	24/S	28/S	25/S	26/S	**13/R**	**0/R**	+

A	A	LMG 22073 *S. acidaminiphila *	30/S	**13/R**	42/S	29/S	*16/I *	42/S	35/S	37/S	33/S	17/S	31/S	28/S	−*
		DSMZ 17805 *S. koreensis *	39/S	37/S	41/S	31/S	40/S	**0/R**	28/S	29/S	32/S	**0/R**	32/S	25/S	−

MR: Multidrug resistance. Amoxicillin/clavulanic acid (AMC); imipenem (IPM); meropenem (MER); ceftazidime (CAZ); cefotaxime (CTX); aztreonam (ATM); ciprofloxacin (CIP); levofloxacin (LVX); chloramphenicol (CHL); trimethoprim-sulfamethoxazole (SXT); tetracycline (TET); tobramycin (TOB). The drug tests were not performed for HIAE 2947, HIAE 4471, LMG 10874, LMG 22074, LMG 25348, *S. pavanii,  *and *S. nitritireducens* strains.

Resistance levels: bold font: resistant; italic font: intermediate; normal font: sensible.

**Table 4:** Nucleotide diversity of the *Stenotrophomonas* spp. analyzed in this study.

Gene	Fragment size (bp)	Polymorphic sites	Mutations		Nucleotide diversity (Pi)
			synonymous	nonsynonymous	
*atp*D (3)	303	24	2	22	0.02013
*gap*A (1)	314	55	48	7	0.03592
*gua*A (3)	401	87	8	79	0.02889
*nuo*D (1)	213	32	28	4	0.02742
*pps*A (1)	257	56	46	10	0.03763
*rec*A (1)	254	67	58	9	0.03749
*rpo*A (1)	136	29	17	12	0.03904

Numbers between parentheses indicate the transcription initiation base position.
